# The Influence of Intravaginal Gestagens Treatment on the Morphological Features and Endometrial Steroid Hormone Receptors Content during Anestrus Type II in Dairy Cattle

**DOI:** 10.3390/ijms23031235

**Published:** 2022-01-22

**Authors:** Michał Trela, Olga Witkowska-Piłaszewicz, Dominika Domańska, Monika M. Kaczmarek, Bartosz Pawliński, Zdzisław Gajewski, Małgorzata Domino

**Affiliations:** 1 Department of Large Animal Diseases and Clinic, Institute of Veterinary Medicine, Warsaw University of Life Sciences, 02-787 Warsaw, Poland; dominika_domanska@sggw.edu.pl (D.D.); bartosz_pawlinski@sggw.edu.pl (B.P.); malgorzata_domino@sggw.edu.pl (M.D.); 2 Department of Hormonal Action Mechanisms/Molecular Biology Laboratory, Institute of Animal Reproduction and Food Research, Polish Academy of Sciences in Olsztyn, 10-748 Olsztyn, Poland; m.kaczmarek@pan.olsztyn.pl; 3 Center for Translational Medicine, Warsaw University of Life Sciences, 02-787 Warsaw, Poland; zdzislaw_gajewsk@sggw.edu.pl

**Keywords:** cow, intravaginal inserts, PR, ER, reproduction, insemination

## Abstract

Background: Gestagens are the most widely used therapy in anestrus type II. The aim of this research is to evaluate the effectiveness of the vaginal progesterone inserts therapy in anestrus type II in cows. Methods: The study was conducted on 33 cows. Progesterone (PR) and estrogen (ER) receptors expression in endometrium was assessed on a molecular level based on mRNA tissue expression. Additionally, blood 17ß-estradiol and progesterone levels were evaluated. Results: A decrease in mRNA expression of A and B PR and ER α was noted in treated and untreated animals. In the treated group, an increase of ERß mRNA expression was observed, while a decreased was found in untreated animals. There was increased PR, ERα and ß expression in endometrial tissue in treated cows, and decreased expression of these factors in untreated cows. In the treated group, recurrence of ovarian cyclicity was noted in 52% of animals and pregnancy was obtained in 34.8% of them, while in the untreated group, recurrence did not occur. In the control group, spontaneous recurrence of ovarian cyclicity was not observed. An increase of PR expression was correlated with increased proliferation of endometrial cells. Conclusions: It seems likely that the endometrium is well developed and ready for placentation after removing the exogenous source of progesterone and preventing the recurrence of cyclicity of ovaries.

## 1. Introduction

The shift toward more productive cows and larger herds is associated with a decrease in reproductive efficiency. The problems contributing to the prolongation of the time from calving to next fertilization are mostly connected with estrus cycle disturbances [1], with occurrences, depending on the research, ranging from 6% to 59%, regardless of insemination [2,3,4], and 20% to 50% after the first insemination [5,6,7]. In well managed herds, intervals to first ovulation in postpartum should take 50–60 days in 85% of cows [8,9]. However, a significant prolongation of this period is a major problem in dairy production. The lack of oestrus or its symptoms, which entail difficulties in effective insemination, increased insemination index, reduced fertilization rates and extended periods of interpregnancy and intercalving service, are called anestrus.

In European countries such as France, as well as in the USA, Canada and China, anestrus occurs in approximately 26.7% of herds [10]. According to the findings of Nation et al. (2001), the prevalence of anestrus in Australian dairy cows is around 19%, while Bartlett et al. (1987) showed a higher percentage in herds in Michigan, i.e., 47% [5,7]. The prevalence of anestrus ranges from 2.1% to 67.1% in India [11], whereas in north-eastern Poland, it is around 26.4% [12]. Thus, the occurrence of anestrus in dairy herds differs not only among the studied regions, but also among herds, which probably is connected with differences in feeding, maintenance, herd management and heat detection [12]. Anestrus in cattle is one of the biggest issues facing the dairy industry, with estimated losses of $300 million in the USA [13]. Reproductive losses in high-producing dairy cattle are connected with prolonged calving intervals, reduced milk production, and increased veterinary costs. In a recent study, optimal economic performance was achieved with a 21-d conception rate, increasing costs by €62.2/cow-year for every 10-percentage-point increase [14].

Under the control of the pituitary hormones such as luteinizing hormone (LH) and follicle-stimulating hormone (FSH), progesterone (P4) plays key role in the uterus [15]. P4 is mostly synthesized by the corpus luteum, but also by the placenta during pregnancy. Thus, the target tissue for P4 and estrogen (E2) is the endometrium. The general regulation of endometrial receptivity for steroid hormones depends on E2 and P4 concentrations. During the follicular phase, high E2 levels upregulate the expression of estrogen (ER) and progesterone (PR) receptors [16], inducing endometrial cell proliferation and expansion of the uterine glands. In contrast, ER and PR downregulation during the luteal phase have been associated with high P4 levels. However, in cows affected by ovarian diseases, P4 and E2 production is disturbed, leading to anestrus.

Currently, anestrus is classified based on the dynamics of growth of ovarian follicles and lutein tissue [17]. This classification is based on the characteristics of the formation of follicles, differentiation and dominance, and clinical symptoms. Four types of anestrus have been distinguished. Type II anestrus, which is examined in this study, is characterized by proper growth and selection of follicles, but ends with atresia of the dominant follicle, which does not ovulate [18]. After 2–3 days, another wave of follicle growth occurs, which also not completed with ovulation. The pathogenesis of type II anestrus is connected with insufficient estradiol synthesis, e.g., as a result of heat stress [19] or increased estrogen clearance, associated with high milk production [20]. Low serum estrogen concentration leads to a decrease of LH level and downregulation of the receptors on the ovaries [21].

The diagnoses in this study are based on rectal ultrasound examinations. The most widely recommended therapy is based on progesterone supplementation [22,23]. However, there is a lack of research on changes in the expression of ER and PR in the endometrium in anestrus type II in dairy cattle. Thus, the aim of this study was to evaluate the expression pattern of these receptors in endometrium in comparison to changes in serum of progesterone and estrogen in dairy cows during anestrus II, and and to determine the influence of progesterone treatment on these parameters.

## 2. Results

### 2.1. Progesterone (P4) Plasma Concentration

**Figure 1:**
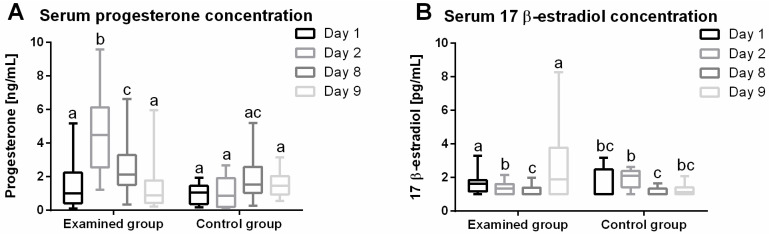
Concentration (minimum value, lower quartile, median, upper quartile, and maximum values) of serum progesterone (**A**) and 17 β-estradiol (**B**) in cow blood on days 1, 2, 8 and 9 of experiment in the study and control groups. In the box plots, the upper whisker represents the maximum value; the upper line of the box represents Q3 (upper quartile); the center line inside the box represents the median; the lower line of the box represents Q1 (lower quartile); and the lower whisker represents the minimum value.

There were significant differences (*p* < 0.0001) in median plasma P4 concentrations (; lower CI; upper CI) during the treatment of anestrus type II with IVD (Figure 1A). In the study group, there was an increase in P4 concentration on day 2 (4.49; 3.67; 5.62 ng/mL) compared to day 1 (1.02; 0.89; 2.08 ng/mL), then a decrease on day 8 (2.12; 1.79; 3.04 ng/mL) versus day 2 (4.49; 3.67; 5.62 ng/mL), and a further decrease on day 9 (0.88; 0.77; 1.99 ng/mL) versus day 8 (2.12; 1.79; 3.04 ng/mL). There were no differences in plasma P4 levels between days 1 (1.02; 0.89; 2.08 ng/mL) and 9 (0.88; 0.77; 1.99 ng/mL). In the control group, there were no significant differences in P4 levels between day 1 (1.05; 0.55; 1.41 ng/mL), day 2 (0.87; 0.43; 1.79 ng/mL), day 8 (1.52; 0.91; 2.88 ng/mL) and day 9 (1.46; 0.99; 2.11 ng/mL).

### 2.2. 17β-Estradiol (E2) Plasma Concentration

Significant differences (*p* < 0.0001) were observed in the concentrations (lower CI; upper CI) of E2 in the blood plasma during treatment of anestrus type II with IVD (Figure 1B). In the study group, a decrease in the concentration of E2 was observed on day 8 (1.00; 1.09; 1.40 pg/mL) in relation to day 1 (1.63; 1.39; 1.88 pg/mL). There was no difference in E2 levels between days 2 (1.36; 1.21; 1.52 pg/mL), 1 and 8. On the day after IVD removal (day 9) (1.89; 1.74; 3.67 pg/mL) an increase in E2 concentration was recorded in comparison to the day before IVD removal (day 8) (1.00; 1.09; 1.40 pg/mL). In the control group, no differences were found between the concentrations of E2 on days 1 (1.00; 1.01; 2.24 pg/mL) and 2 (2.11; 1.53; 2.36 pg/mL), while a decrease was found on day 8 (1.00; 0.96; 1.35 µg/mL) versus day 2 (2.11; 1.53; 2.36 pg/mL).

### 2.3. Progesterone Receptors (PR) mRNA Expression in Endometrium

**Figure 2:**
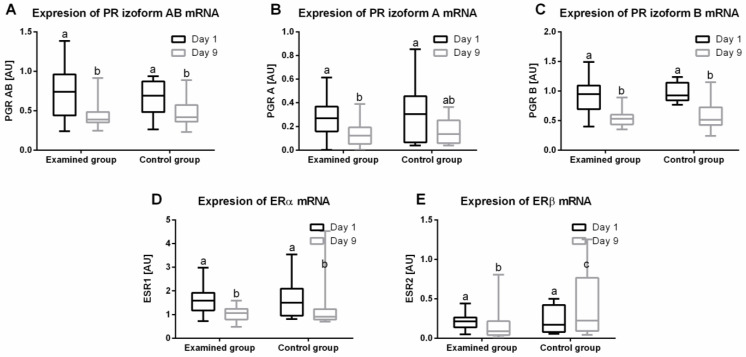
Endometrial expression (minimum value, lower quartile, median, upper quartile, and maximum values) of AB isoform PR (**A**), A isoform PR (**B**), B isoform PR (**C**), ERα (**D**), ERβ (**E**) mRNA on days 1 and 9 of the experiment in the study and control groups. In the box plots, the upper whisker represents the maximum value; the upper line of the box represents Q3 (upper quartile); the center line inside the box represents the median; the lower line of the box represents Q1 (lower quartile); and the lower whisker represents the minimum value.

**Figure 3:**
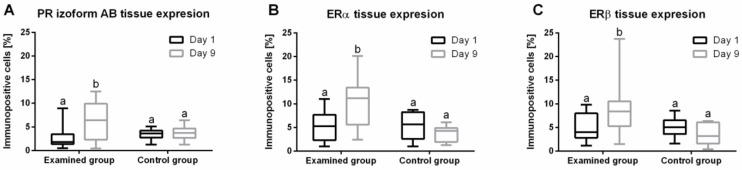
Endometrial expression (minimum value, lower quartile, median, upper quartile, and maximum values) of PR AB (**A**), ERα (**B**), ERβ (**C**) proteins on day 1 and day 9 of experiment in the study and control groups. In the box plots, the upper whisker represents the maximum value; the upper line of the box represents Q3 (upper quartile); the center line inside the box represents the median; the lower line of the box represents Q1 (lower quartile); and the lower whisker represents the minimum value.

There were significant differences in the expression (median; lower CI; upper CI) of all isoforms of PR mRNA in the endometrium: AB (*p* = 0.0002) (Figure 2A), A (*p* = 0.0052) (Figure 2B) and B (*p* < 0.0001) (Figure 3C). In the study group, there was a decrease in the expression of PR mRNA on day 9 for AB (0.39; 0.37; 0.50 AU), A (0.12; 0.09; 0.20 AU), and B (0.53; 0.49; 0.60 AU) isoforms vs. day 1 AB (0.74; 0.60; 0.85 AU), A (0.27; 0.20; 0.33 AU) and B (0.95; 0.80; 1.06 AU). In the control group, there was a decrease in the expression of PR mRNA on day 9 of both isoforms, as measured by one assay AB (0.42; 0.33; 0.61 AU) and B isoform (0.51; 0.39; 0.80 AU) (*p* = 0.0014), compared to day 1: AB (0.69; 0.50; 0.83 AU) and B (0.98; 0.86; 1.10 AU). There were no changes in the expression of isoform A on day 9 (0.14; 0.08; 0.24 AU) vs. day 1 (0.31; 0.12; 0.50 AU).

### 2.4. Estrogen Receptors (ER) mRNA Expression in Endometrium

There were significant differences in the expression (median; lower CI; upper CI) of ER mRNA in the endometrium: ERα (*p* < 0.0001) (Figure 2D) and ERß (*p* < 0.0112) (Figure 2E) during the treatment of anestrus type II with IVD. In the study group, after therapy, a decrease in the expression of the ERα mRNA was noted on day 9 (1.06; 0.91; 1.17 AU) in relation to day 1 (1.59; 1.39; 1.84 AU). In addition, in the control group, there was a decrease in the expression of the ERα on day 9 (0.91; 0.48; 2.13 AU) compared to day 1 (1.50; 1.04; 2.30 AU). In the study group, a decrease in the expression of ERβ mRNA was noted on day 9 (0.09; 0.08; 0.24 AU) in relation to day 1 (0.21; 0.17; 0.26 AU). Moreover, in the control group, there was an increase in the expression ERβ mRNA on day 9 (0.22; 0.09; 0.74 AU) compared to day 1 (0.17; 0.10; 0.34 AU).

### 2.5. Progesterone Receptors (PR) Protein Expression in Endometrium

During the treatment of anestrus type II with IVD, significant differences were noted in the tissue expression (median; lower CI; upper CI) of the PR AB isoform in the cows’ endometria (*p* = 0.0004) (Figure 3A). In the study group, there was an increase in the PR AB tissue expression on day 9 (6.40; 4.64; 7.85) compared to day 1 (1.80; 1.80; 3.62). In the control group, there were no differences in the PR AB tissue expression between days 1 (3.64; 2.64; 4.25) and 9 (3.74; 2.70; 4.78). Moreover, there were no differences in the PR AB tissue expression on day 1 between the study groups (1.80; 1.80; 3.62 vs. 3.64; 2.64; 4.25).

### 2.6. Estrogen Receptors (ER) Protein Expression in Endometrium

There were significant differences in ER tissue expression in the endometrium during the treatment of anestrus type II with IVD; for ERα (*p* = 0.0002), see Figure 3B; and for ERß (*p* = 0.0007), see Figure 3C. The test group showed an increase in the tissue expression (median; lower CI; upper CI) of ERα on day 9 (11.18; 8.06; 12.30) compared to day 1 (5.27; 3.95; 6.57). In the control group, no significant differences were reported in ERα tissue expression at days 1 (5.66; 3.22; 7.27) and 9 (4.29; 2.46; 4.91). Additionally, there were no significant differences (*p* > 0.05) in the expression of the ERα tissue expression between the study groups on day 1: test (5.27; 3.95; 6.57) and control group (5.66; 3.22; 7.27). In the study group, there was an increase in ERβ tissue expression on day 9 (8.41; 7.20; 11.84) compared to day 1 (4.04; 3.79; 6.26). In the control group, no differences in ERβ tissue expression between days 1 (5.06; 3.67; 6.54) and 9 (3.53; 1.94; 5.12) were reported. Moreover, there were no differences between the examined groups on day 1: study (4.04; 3.79; 6.26) and control (5.06; 3.67; 6.54).

### 2.7. Histological Evaluation of Endometrium Reaction

**Figure 4:**
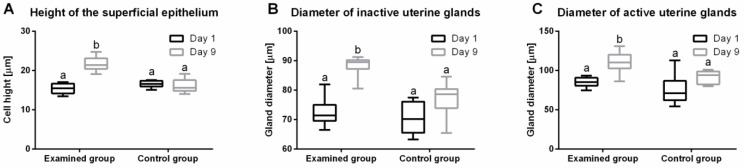
Endometrial measurements (minimum value, lower quartile, median, upper quartile, and maximum values) of height of the superficial epithelium (**A**), diameter of inactive uterine glands (**B**) and diameter of secretionally active uterine glands (**C**) on days 1 and 9 of the experiment in the study and control groups. In the box plots, the upper whisker represents the maximum value; the upper line of the box represents Q3 (upper quartile); the center line inside the box represents the median; the lower line of the box represents Q1 (lower quartile); and the lower whisker represents the minimum value.

**Figure 5:**
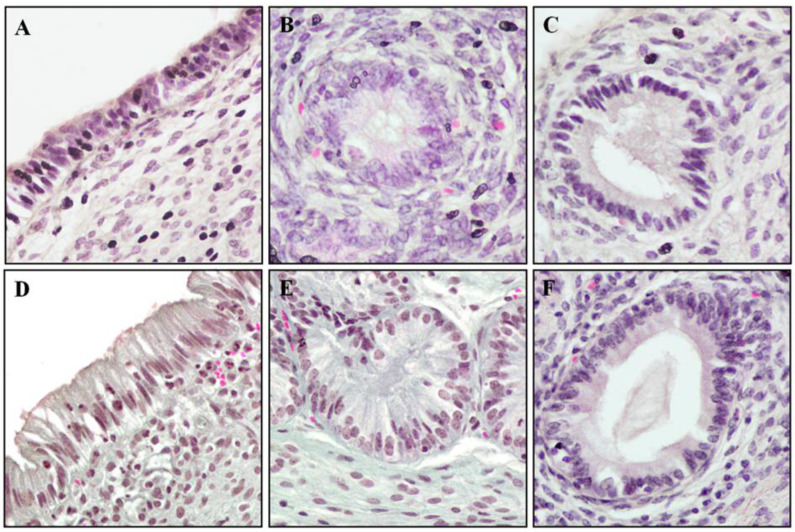
Tissue endometrial reaction in the study group in response to progesterone therapy of anestrus in type II. H&E staining, light microscope, magnification 40×. Superficial epithelium on days 1 (**A**) and 9 (**D**); nonactive uterine glands on days 1 (**B**) and 9 (**E**); active uterine glands on days 1 (**C**) and 9 (**F**).

During treatment of anestrus type II with IVD, significant differences were noted in tissue responses (median; lower CI; upper CI), height of the superficial epithelium (*p* = 0.04) (Figure 4A), the diameter of inactive uterine glands (*p* = 0.06) (Figure 4B) and the diameter of active uterine glands (*p* = 0.03) (Figure 4C). In the study group on day 9, there was an increase in the height of the superficial epithelium (21.4; 21.0; 22.4 μm) and the diameters of inactive glands (89.5; 87.3; 89.5 μm) and active glands (110.0; 105.2; 115.6 μm) compared to day 1 (15.4; 15.0; 16.0 μm, 71.4; 70.3; 73.7 μm and 85.3; 82.9; 88.2 μm, respectively (Figure 5). In the control group, no differences were reported: superficial epithelium (day 1: 16.6; 15.9; 17.2 μm; day 9: 15.6; 14.8; 17.2 μm), the diameter of inactive glands (day 1: 70.1; 66.6; 74.7 μm; day 9: 78.6; 73.3; 81.0 μm) and active glands (day 1: 71.1; 62.1; 89.9 μm; day 9: 94.1; 86.5; 97.7 μm).

### 2.8. Correlations

In the study group, on day 1, before starting therapy with IVD, a strong, directly proportional correlation was found between the PR AB and PR B mRNA expression (Sp = 0.75; *p* = 0.0003) which was directly proportional with the PR AB mRNA expression and mRNA ER α (Sp = 0.52; *p* = 0.01) and PR A and B mRNA expression (Sp = 0.54; *p* = 0.007). On day 9, after removal of the IVD, a directly proportional correlation was found between PR AB and B mRNA expression (Sp = 0.57; *p* = 0.004) and PR A and B mRNA expression (Sp = 0.62; *p* = 0.001). There was also an average, directly proportional relationship between PR AB mRNA expression and tissue ER α expression (Sp = 0.53; *p* = 0.008). In the control group, on day 1, before the initiation of therapy, strong, directly proportional correlations were found between PR AB and B mRNA (Sp = 0.64; *p* = 0.04), as well as between PR AB and the A mRNA expression (Sp = 0.74; *p* = 0.012). There was also a strong, directly proportional correlation between peripheral P4 and the ER α mRNA expression (Sp = 0.67; *p* = 0.04) and an average, inversely proportional correlation between peripheral P4 and ER ß mRNA expression (Sp = −0.51; *p* = 0.003).

On day 9, in the period of the removal of the IVD in the study group, strong, directly proportional correlations were found between PR AB and B mRNA expression (Sp = 0.64; *p* = 0.04) and PR A and B mRNA expression (Sp = 0.61; *p* = 0.003). There was also a strong, directly proportional relationship between peripheral P4 and ER α mRNA expression (Sp = 0.67; *p* = 0.04) and tissue PR AB expression (Sp = 0.70; *p* = 0.03). A negatively proportional correlation was also demonstrated between PR AB mRNA and ß ER mRNA expression (Sp = −0.53; *p* = 0.008).

## 3. Discussion

P4 treatment influences PR mRNA and tissue expression. In one study, PR mRNA expression increased from the ovulation phase, reaching a maximum level on the 6–8th cycle day before starting to decrease on day 12 [24]. In a study performed by Kimmins and MacLaren (2001) [25], the maximum PR mRNA expression was recorded between the 1st and 6th days of the estrus cycle. However, in other studies, the highest PR expression was recorded between days 1 and 5 and 17 and 20, and the lowest between days 6 and 16 of the cycle [26]. In our study, P4 therapy induced the expression of its own receptors in endometrial tissue, whereas mRNA expression was downregulated in anestrus type II. This was probably connected with the short lifespan of mRNA and/or transcription and translation processes leading to the synthesis of proteins [27]. The activation of miRNAs and other proteins determines the fate of any mRNA, and by extension, their expression and degradation, as has been confirmed in other animals studies [28]. Moreover, mRNA degradation plays a key role in the direct control of gene expression [29]. Therefore, it can be assumed that the regulation of PR and ER expression at the nuclear level takes place before day 9 of the progesterone dominance phase, although its role in the treatment of type II anestrus requires further studies. Due to the effective increase in PR expression and tissue reaction, as well as the high rate of pregnancies, it can be concluded that a significant decrease in mRNA expression in the tested receptors, visible in the second sampling of the endometrium, is a normal, regulatory element.

In addition, P4 acts on target cells through membrane receptors which exist in two main isoforms: A and B. PR B is a progesterone-dependent gene activator, while PR A is a potent inhibitor of PR B that reduces the effects of P4 in tissue [30]. Thus, higher tissue expression of PR increases the sensitivity of the endometrium, and can stimulate the proliferation of endometrial cells [31]. Therefore, the concomitant increase in tissue AB PR expression and the endometrial response to hormonal stimulation may ensure high pregnancy rates in the first insemination following treatment. Thus, we confirmed that the expressions of PR A and B in tissue influence each other, and are regulated on a nuclear level. In the control group, there was decrease in mRNA A, B, and AB PR expression which did not influence tissue PR expression.

During the follicular phase, high peripheral E2 levels via ER induce endometrial cell proliferation, expansion of the uterine glands and PR mRNA transcription. The gradually increasing endometrial sensitivity to P4 peaks in the luteal phase with low peripheral E2 levels and high P4. In our study, E2 plasma concentrations in treated group decreased significantly after insertion of an IVD until the day of its removal (day 8). At the same time, the low E2 peripheral concentration influenced ER α and β expression at the mRNA endometrial level. The significant decreases in ER α and β mRNA expression were mostly visible in the second sampling (on day 8). This confirmed poor mucosa sensitivity and response to estrogens. In another study, the highest expression of ER mRNA was recorded during estrus and in the early luteal phase (days 4–10 of the cycle), the lowest between day 12 and 16 of the cycle, whereas another increase was noted after day 16 [24]. Additionally, in ovariectomized animals, low ER expression following P4 administration, as well as P4 together with E2, were confirmed [25]. Since ER tissue expression is under the control of P4 and E2 [32], it can be assumed that in the absence of estrogen stimulation, ERs are regulated by P4. Progesterone primarily inhibits the tissue ERα expression to prevent the excessive proliferation of endometrial cells [33], but in the face of peripheral E2 deficiency, it also stimulates ER expression, thus increasing tissue sensitivity to E2 [18,34].

However, in the cows from untreated group, there was a significant increase in ER β mRNA expression on day 9 compared to day 1. This result is interesting, due to ER β activation, which blocks ER α gene transcription in the normal cycle [35]. Thus, it reduces the growth of the uterine mucosa, which additionally translates into a reduction in the endometrial response to hormonal changes. The lack of differences in the height of the superficial epithelium, the diameter of the glandular epithelium and the secretion of the uterine glands was confirmed in our study. Thus, it may result in failed pregnancies after insemination. In the control group, no estrus symptoms were found in the tested cows. Thus, the increase in ER mRNA expression may translate into an additional limitation of the growth of the uterine mucosa in the period preceding insemination.

An increase in plasma E2 concentrations were recorded following the growth of the follicles on day 9 (1 day after IVD removal). These observations are probably related to the restoration of ovarian cyclic activity as a result of the “rebound effect”, which is induced after removal of the external source of P4, and to the influence of the hypothalamic-pituitary-ovary axis. This increased the percentage of endometrial cells expressing ER α and β after P4 therapy. In the control group, no changes in ER tissue expression were noted throughout the experiment. Robinson et al. (2001) found the maximum ER tissue expression in estrus and in the middle of the luteal phase (days 10–14 of the cycle) in cyclic cows, and the minima around the 6th and 16th days of the cycle, as well as an increase in the next follicular phase, which is in line with our findings [36].

In this study, we confirmed that increased P4 peripheral level influences PR, ER α and ER ß tissue expression. Thus, it influences endometrial sensitivity to P4 and E2. Endometrial PR mRNA is expressed during the estrus cycle mainly in the subepithelial stroma and superficial glands, and, to a much lower extent, in the superficial epithelium [37,38]. Additionally, estrogen-dependent regulation of endometrial cell proliferation and differentiation takes place both directly, through ER α activation, and indirectly, through the insulin-like growth factor (IGF) pathways in the subepithelial stroma [39]. In our study, based on histological evaluations, the proliferation rates of target cells were most pronounced in these areas. The endometrial reaction of the epithelium, glandular epithelium and uterine gland secretion may lead to increased numbers of pregnancies. However, more studies are needed, because successful inseminations and maternal recognition of pregnancy take place in ongoing cycles. The endometrial states in the cycles after treatment remain unknown. In this study, an increase of plasma P4 at the beginning of treatment occurred, while a decrease on day 8 was noted. However, supplementation by IVD is a local process which mostly effects endometrial P4 concentrations, as confirmed by histological evaluation.

In this study, the effect of P4 therapy on the restoration of ovarian activity in type II anestrus and the possibility of obtaining and maintaining pregnancy immediately after treatment in cattle were evaluated. The present study confirmed the effectiveness of anestrus type II therapy in cattle with the IVD [23]. In addition, this therapy is considered to be the most effective available treatment for anestrus in cattle [40]. A meta-analysis based on 25 publications from 2004–2015, which evaluated a total of 8285 cows treated with different estrus induction or synchronization protocols using progesterone-containing inserts, showed that the administration of P4 during the follicle growth wave significantly increased fertility rates, as did P4 supplementation during Ovsynch programs, which increased their effectiveness by about 10% and reduced the risk of pregnancy loss [22]. In the present study, the effectiveness of restoring estrus using P4 inserts in anestrus type II in cattle was 52% with a fertilization rate of 34.8%. Our findings did not differ significantly from those of other studies [41,42].

## 4. Limitations

The main limitation of this study was its relatively small sample size. However, we tried to avoid an influence of environmental factors, using cows from a single one farm. Another limitation was that we performed the study only during and just after progesterone treatment. Based on good ethical practices in performing experiments in animal models, we avoided in vivo studies in the biggest group for any longer than necessary. While designing the experiment protocols, we tried to achieve the most suitable conditions to obtain the most accurate results possible.

## 5. Materials and Methods

The research was conducted with the consent of the 3rd Local Ethical Committee for Experiments on Animals in Warsaw (No. 5/2014).

### 5.1. Animals

Thirty-three cows with anestrus type II aged 2–8 years (1–5 lactation), weighing 550–650 kg, BCS 3.0–3.5 and between 60 and 125 days of lactation were selected from 350 cows from a high-producing dairy herd. Animals were randomly assigned to the study (*n* = 23) and control groups (*n* = 10). No clinical symptoms of other disorders of the reproductive system or metabolic disturbances were observed. The animals were kept in a stand-alone system, fed ad libitum with complete formula (TMR), with milking three times a day. During the experiments, the animals were under constant veterinary care. A clinical examination was performed prior to each sampling. Throughout the experiment and after its completion, the animals remained in the production group, with a mean daily milk yield of 31.9 kg milk/cow).

### 5.2. Therapy Protocol

**Figure 6:**
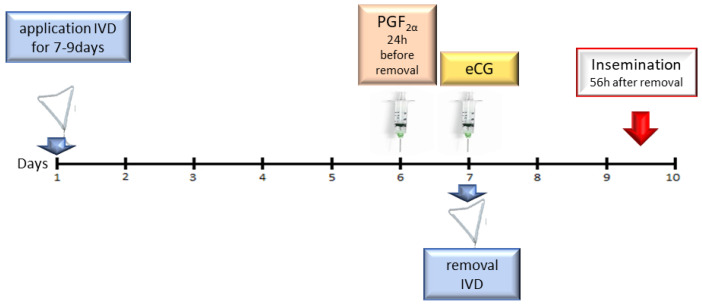
Treatment protocol.

On the first day of the experiment, an intravaginal progesterone releasing device (IVD) (PRID Delta^®^, Ceva Sante Animale S.A., Libourne, France) containing 1.55 g of natural progesterone (P4) was inserted in to examination group (van Werven 2013; Silva 2021). On day 7, prostaglandin F2α (PGF2α) (dinoprost 25 mg/animal, Enzaprost 5 mg/mL, Ceva Sante Animale S.A., Libourne, France, i.m.) was administered to all cows. On day 8, IVDs were removed and eCG was administered at a dose of 500 IU/animal (Syncrostim^®^, Ceva Sante Animale S.A., Libourne, France, i.m.). After that, estrus symptoms were observed and insemination was performed 56 h later (Figure 6).

The control group received no treatment.

### 5.3. Sampling

Blood samples in both groups were collected four times: on day 1 (before insertion of the IVD), day 2 (the day after application of the vaginal insert), day 8 (after removal of the insert) and day 9 (the day after application of the insert). Blood samples were taken from the tail vein (Vena coccygea mediana, s. Vena caudalis mediana) directly into 7.5 mL EDTA tubes (KABE Labortechnik, Nümbrecht, Germany). After collection, blood was stored at 4 °C and centrifuged for 10 min (5000 rpm, 3354× *g*) within one hour of collection to obtain 4 mL of plasma. Plasma was then frozen in 1.5 mL Ependorff tubes at −20 °C. The material was stored at −20 °C until the concentrations of progesterone and 17β-estradiol were determined.

Biopsy samples were taken on day 1 (before application of vaginal inserts) and day 9 (the day after the application of vaginal inserts). Endometrial biopsies were performed using Eppendorf forceps, modified according to our own design, in the area of the transition of the body to the uterine horn. The site of collection of the material was manually controlled by rectal examination.

### 5.4. Analyses of mRNA Expression in Endometrium

Total RNA was extracted using the TRI Reagent (Invitrogen, Thermo Fisher Scientific, USA) and BCP (Molecular Research Center Inc., Cincinnati, OH, USA). For RNA purification, the PureLink RNA Mini Kit (Invitrogen, Thermo Fisher Scientific, USA) was used. Genomic DNA was removed using the PureLink^®^ DNase Set (Invitrogen, Thermo Fisher Scientific, Waltham, MA, USA). The total RNA was stored at −80 °C in nuclease-free water with the addition of an RNAz inhibitor (Applied Biosystems, Thermo Fisher Scientific, Waltham, MA, USA). The amount and purity of the obtained RNA was assessed using a NanoDrop 1000 spectrophotometer (Thermo Fisher Scientific, Waltham, MA, USA).

A High Capacity cDNA Reverse Transcription kit (Applied Biosystems, Thermo Fisher Scientific, Waltham, MA, USA) was used to transcribe 1 µg or 500 ng of RNA (for low concentration assays) into cDNA, following the manufacturer’s instructions. The reaction mixture contained 10 × RT buffer, 100 mM 25 × dNTP mix, 10 × RT random primers, 1 U/µL RNAse inhibitor, and MultiScribe ™ Reverse Transcriptase. The use of the reaction mixture without the reverse transcriptase enzyme and the replacement of the RNA template with water allowed us to perform control reactions. The reverse transcription reaction was performed in a thermocycler (SensoQuest, Göttingen, Germany) under the following conditions: 10 min at 25 °C, 120 min at 37 °C and for 5 min at 85 °C.

TaqMan^®^ Gene expression Master Mix reagents and 20 × TaqMan^®^ Gene Expression Assays were used for real-time PCR reactions (Thermo Fisher Scientific, Waltham, Massachusetts, USA). The expression of the following genes was examined: *ESR1 (Bt03210039_m1), ESR2 (Bt03259200_m1)*, *PGAB* (Custom Plus TaqMan Assay, [37] and *PGB* (Custom Plus TaqMan Assay), [43], as well as reference genes *GAPDH (Bt03210913_g1)*, *ACTB (Bt03279174_g1)* and *HPRT (Bt03225305_g1)*. The reaction mixture (10 μL) contained 3 μL of diluted cDNA (15 ng), 5 μL of 2 × TaqMan^®^ Gene expression Master Mix, 0.5 μL of 20 × TaqMan^®^ Gene Expression Assays, and 1.5 μL of nuclease-free water (Applied Biosystems, Thermo Fisher Scientific, Waltham, MA, USA). The amplification reaction was performed in duplicate on an ABI Prism 7900 HT thermocycler (Applied Biosystems, Thermo Fisher Scientific, Waltham, MA, USA) under the following conditions: incubation at 50 °C for 2 min, initial denaturation for 10 min at 95 °C, followed by 45 cycles of denaturation for 15 s at 95 °C and primer annealing/strand extension for 1 min at 60 °C. The Ct values obtained during real-time PCR analyses were corrected using the Real-time PCR Miner program [43]. The qPCR efficiency and CT values for individual reactions were determined by analysis of raw fluorescence data [43]. NormFinder software was used to select the most stable reference genes.

All expression data for each gene were divided by the geometric mean of two of the most stable reference genes in the endometrium, i.e., ACTB and HPRT (with a stability value = 0.167), and expressed as arbitrary units [AU]. The value of PGA expression (encoding PR A) was obtained by subtracting expression isoform B from AB (PGAB and PGB) in relation to those of housekeeping genes. These data are presented in the form of index values of selected indexes ± standard deviation.

### 5.5. Measurements of the Systemic Concentration of 17 ß-Estradiol and Progesterone

The radioimmunological method (RIA) was used to determine the concentrations of ovarian steroid hormones 17 ß-estradiol (E2) and progesterone (P4) in the blood plasma. The sensitivity of the RIA for E2 concentrations (ESTRUS-US-CT, CIS BIO ASSAYS, France) was 1.36 pg/mL (curve range 2.72 pg/mL to 550 pg/mL). The sensitivity of the RIA for P4 determination (Diasource Cat # KIP 1458, DIAsource ImmunoAssays S.A., Ottignies-Louvain-la-Neuve, Belgium) was 0.05 ng/mL (curve range 0.12 ng/mL to 36.0 ng/mL).

### 5.6. Endometrial Measurements

Slides were stained with the hematoxylin-eosin (HE), according to standard procedure (Aqua-med, Tarnoberzeg, Poland). The prepared slides were assessed under a light microscope (BX-61, Olympus Polska Sp.z o.o., Warsawa, Poland). Then, a basic histometric analysis was performed using the CellSens Standard Image Analysis software (Olympus Polska Sp.z o.o., Warsaw, Poland) in order to determine the height of the superficial epithelium, the diameter of inactive uterine glands and the diameter of the secretory-active uterine glands.

Immunofluorescent staining was performed according to the standard procedure [44]. For the visualization of the ER α, ER β and PR of the AB isoform, the following specific primary mouse monoclonal antibodies were used: mouse antihuman estrogen receptor alpha (Bio-Rad Laboratories, Warszawa, Poland), mouse antihuman estrogen receptor beta 1 (Bio-Rad Laboratories, Poland) and mouse antihuman progesterone receptor 1AB (ProGen, Heidelberg, Germany). Secondary antibodies conjugated with the fluorochrome AF 568 (Alexa Fluor 568 donkey antimouse; Invitrogen, USA) or AF 633 (Alexa Fluor 633 goat antimouse; Invitrogen, Thermo Fisher Scientific, Waltham, MA, USA) were used to visualize the primary reaction. Cell nuclei were stained with Heochst 33,342 intercalating with DNA (200 µL; concentration 0.02 mg/mL; Sigma-Aldrich, Darmstadt, Germany). The Fluoromount Aqueous Mounting fluorescence visualization medium (Sigma-Aldrich, Darmstadt, Germany) was used. The preparations were assessed in a scanning cytometer (SCAN ^ R, Olympus Polska Sp.z o.o., Warsaw, Poland), and with a confocal microscope (FV-500, Olympus Polska Sp.z o.o., Warsaw, Poland).

Regarding confocal microscopy, the specificity of the antigen-antibody reaction was assessed to visualize the AB isoform PR, ER α and ER β, respectively. In contrast, the expression of PR AB, ER α and ER β was assessed in a scanning cytometer.

### 5.7. Statistical Analysis

All results were reported means + SD. All statistical analyses were performed using the GraphPad Prism6 software (GraphPad Software Inc., San Diego, CA, USA). Numerical data are reported in box plots using minimum and maximum values, lower and upper quartiles as well as medians.

The compatibility of distributions on the testing days with the normal distribution were analyzed using a univariate Kolmogorov-Smirnov test, with α = 0,05. Differences between the groups and subsequent samples were considered significant at *p* < 0.05. Hormonal profiles were tested using one-way analysis of variance (one-way ANOVA) assessed by Kruskal–Wallis test, followed by Dunn’s multiple comparisons due to the non-Gaussian data distribution. Changes in progesterone and 17ß-estradiol concentrations were considered separately.

The expression of ESR1, ESR2, PGAB, PGA, PGB genes, and protein expression of ER α, ER ß, PR AB were analyzed using one-way analysis of variance (one-way ANOVA) assessed by Kruskal–Wallis test, followed by Dunn’s multiple comparisons test for evaluate differences between the groups. A Mann-Whitney’s test or Welch’s *t*-test was used to evaluate differences between subsequent sampling collections, depending on the data distribution.

Spearman’s rank correlation coefficient was calculated for all analyses of hormone levels, mRNA expression and tissue expression, irrespective of the group (on days 1 and 9). Correlations were significant at *p* < 0.05.

## 6. Conclusions

In type II anestrus in cattle, the expression of receptors in the endometrium (ER, PR) is disturbed. Low level of plasma E2 concentrations lead to insufficient endometrial hyerplasia. Estrogen-dependent transcription of PR mRNA is also limited. Therefore, this may results in low numbers in pregnancies at first ovulation and spontaneous ovarian cycle activity recovery. In addition, high peripheral P4 concentrations can stimulate the proliferation of endometrial cells. When the exogenous source of P4 is removed and the cycle of the ovaries is restored, the endometrium is prepared for embryo implantation. Therefore, the rates of pregnancy after insemination at the first ovulation after anestrus type II progesterone therapy are significantly higher compared to spontaneous recovery.

## Data Availability

The data presented in this study are available on request from the corresponding author.
